# Sartorius and Gracilis Muscle Flaps as Adjuncts for the Management of Complicated Femoral Wounds in Vascular Surgery

**DOI:** 10.1016/j.ejvsvf.2025.03.004

**Published:** 2025-03-18

**Authors:** Jhanvi Dholakia, Anantha Narayanan, Qiantai Hong, Manar Khashram

**Affiliations:** a Department of Vascular Surgery, Waikato Hospital, Hamilton, New Zealand; b Department of Surgery, University of Auckland, Auckland, New Zealand

**Keywords:** Gracilis flap, Groin reconstruction, Surgical wound, Wound healing

## Abstract

**Aim:**

Groin complications following vascular surgery occur in 10–30% of cases and are associated with significant morbidity and mortality rates. While various institutions have published results on sartorius and rectus femoris muscle flaps for arterial reconstruction coverage, there are limited data on the use of gracilis muscle flaps. This study aimed to present the outcomes of sartorius and gracilis muscle flaps in managing complicated femoral wounds.

**Method:**

A retrospective study was conducted from January 2019 to December 2023 at a tertiary centre in New Zealand. The study design followed the STrengthening the Reporting of Observation studies in Epidemiology (STROBE) checklist. Patients who underwent a muscle flap for an emergency vascular surgery indication were included. The primary outcome was wound healing, while secondary outcomes included re-intervention, limb and graft salvage.

**Results:**

Twenty-three flaps were performed for post-operative complications during the study period, including 10 gracilis flaps and 13 sartorius flaps, with a median follow up of twenty-six months. Complete healing was achieved in 90% of patients in the gracilis group, compared with 69% in the sartorius group (*p* = 0.34). The median time to healing was 56 days in the gracilis group and 55 days in the sartorius group. Three patients in the sartorius group underwent planned re-intervention for groin debridement, compared with two in the gracilis group. Donor site wound complications occurred in two gracilis flap patients, both of which healed. Major complications in the sartorius group included two amputations and one graft occlusion, while no such event occurred in the gracilis group.

**Conclusion:**

Both gracilis and sartorius muscle flaps are viable options for reconstructing femoral wounds, with reasonable post-operative outcomes. Further multicentre studies are needed to better correlate clinical outcomes with the perceived benefits of the gracilis flap.

## INTRODUCTION

An infected femoral wound following vascular reconstruction is a challenging and potentially devastating occurrence, with an incidence ranging 10–30%.^1^^,^^2^ Management can be associated with significant morbidity in the form of complex wound care, lower quality of life, graft or limb loss, and death.^3^ While superficial groin infections can be managed with non-operative measures,^4^^,^^5^ deep infections and dehiscence may require extensive debridement to gain source control, leading to exposure of the underlying vasculature. In these situations, muscle flaps can be a useful tool in the armamentarium to facilitate soft tissue coverage and graft salvage.

Sartorius muscle flaps (SMFs) and rectus femoris muscle flaps (RFFs) have traditionally been used for groin coverage.^6^, ^7^, ^8^, ^9^ Due to their proximity to the femoral vessels and segmental blood supply, SMFs are classically favoured; however, the primary blood supply of the muscle is the superficial femoral artery, which is often affected in patients with end stage peripheral artery disease.^10^, ^11^, ^12^ The benefit of RFFs is their larger muscle bulk and ability to aid reconstruction of the inguinal ligament, but they are related to increased donor site morbidity.^13^, ^14^, ^15^ Due to the considerable muscle bulk and favourable arc of rotation, gracilis muscle flaps (GMFs) have emerged as a third option. Furthermore, GMFs derive their blood supply from the profunda femoris artery and are associated with fewer donor site complications.^16^, ^17^, ^18^ Previous studies have been unable to adequately compare outcomes for SMFs with GMFs due to small numbers of gracilis flaps being performed.^12^

Thus, this study aimed to directly present outcomes following SMFs or GMFs in a single centre cohort of patients with deep groin wound infections following vascular reconstruction.

## METHOD

### Study

A retrospective cohort study was carried out between January 2019 to December 2023 at Waikato Hospital. The STrengthening the Reporting of OBservational studies in Epidemiology (STROBE) checklist was used in the design and reporting.^19^ Patients who underwent SMF or GMF reconstruction for groin wound closure over a vascular reconstruction by vascular surgeons in the expedited setting were identified through the hospital database. Patients who had died prior to wound healing were considered unhealed. Follow up was carried out by the vascular team in the outpatient setting along with district nursing in the community. A retrospective chart review was carried out until July 2024. Written consent for the use of patient details and outcomes for the purposes of research was obtained at the time of surgery.

### Variables

Patient demographics, comorbidity data, immunosuppression use (steroid, immunomodulators, current chemotherapy), and smoking status were recorded from a patient chart review. Comorbidities of interest included type 2 diabetes mellitus (HbA1c > 48 mmol/mol), recorded history of hypertension, ischaemic heart disease (previous myocardial infarction), and chronic kidney disease. Pre-operative imaging was used to calculate a standardised femoral artery depth, as described by Durand *et al.*, as a surrogate measure of body mass index.^20^ The American Society of Anaesthesiologists (ASA) classification was recorded as a cumulative measure of overall patient physiological status. Graft types were recorded as being autologous (native vessel, vein grafts) or prosthetic (bovine pericardium, polyester, polytetrafluoroethylene). The primary endpoint was complete and closed wound healing with intact skin, free of infection. The secondary outcomes were time to wound healing, expected or unexpected re-intervention (expected re-intervention such as debridement and application of dressing; unexpected re-intervention for flap necrosis or graft explantation or donor site complications), limb loss and graft patency. Data were also recorded on wound specimen bacterial culture, antibiotic choice, and total antibiotic duration.

### Surgical technique

#### Sartorius

**Figure 1:**
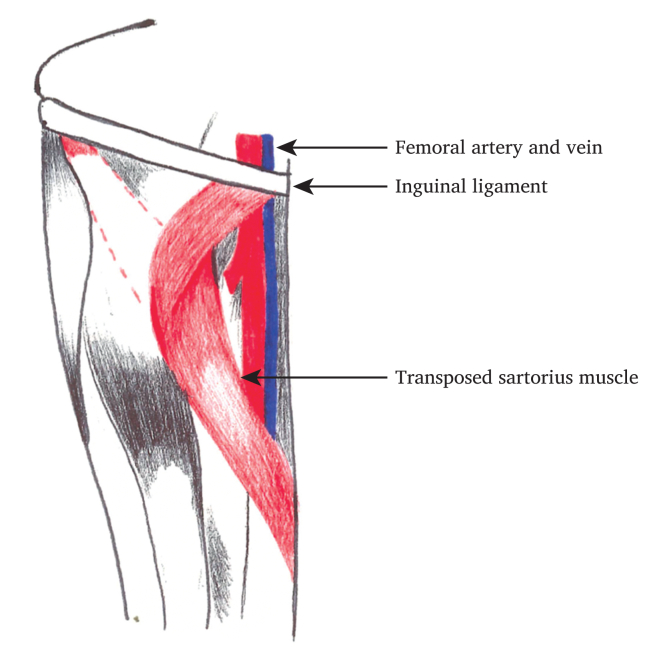
Depiction of sartorius flap formation.

The sartorius muscle lateral to the femoral neurovascular bundle is identified and mobilised, keeping the vascular supply from its medial border intact. The muscle attachment is divided at the anterior superior iliac spine. The muscle flap is rotated medially and secured to the inguinal ligament to cover the groin defect (Fig. 1).

#### Gracilis

**Figure 2:**
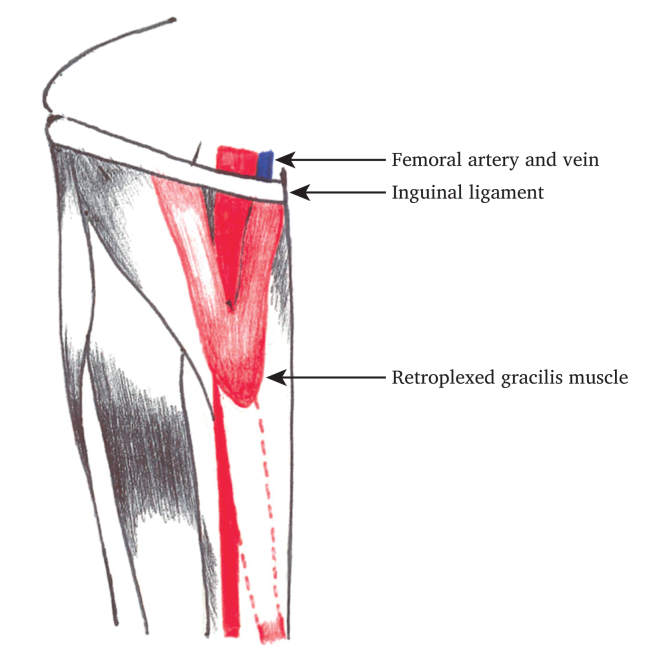
Depiction of gracilis flap formation.

The tapering distal gracilis muscle is tendinous and contains no perforator vascular supply. A 7–10 cm incision is made in the medial distal thigh, the tendinous attachment is dissected at its most distal aspect and reflected upwards or plexed to cover the defect, by suturing it to the inguinal ligament. The medial circumflex artery branch is left intact (Fig. 2).

While the study was retrospective, there were factors that influenced the choice of whether patients had a GMF or SMF. If patients were undergoing a medial thigh incision for saphenous vein harvest or exposure of supragenicular vessels, creating a GMF was preferred. In patients with an existing femoropopliteal or distal graft, a primary SMF was preferred, as mobilisation of gracilis requires more extensive dissection in scarred tissue planes and it may compress the graft if attempting to retroplex the gracilis. A GMF was preferred for patients with multiple revision groin surgery, as the sartorius muscle is more likely to be scarred. An SMF was preferred for patients where the groin wound is large and requires extensive debridement, as the tissue planes have already been identified and dissected. While the authors have not experienced this, this approach would allow for the creation of a future GMF should the SMF fail.

### Statistical analysis

Categorical variables are reported as frequency and continuous variables as median. Statistics were calculated using a two tailed *t* test for parametric data, and the Mann–Whitney *U* test for non-parametric data. Factors associated with the primary outcome were analysed using multiple regression analysis. A subset analysis was carried out on the re-intervention group and limb loss group. *P* values <0.05 were considered to be statistically significant. All statistical analysis was carried out using SPSS (IBM, Armonk, NY, USA).

### Ethics approval

This project was deemed out of scope by the New Zealand Health and Disability Ethics Committee (HDEC REF: 2024 OOS 20857).

## RESULTS

### Demographics

There were 23 patients who underwent muscle flaps for groin coverage between 2019 and 2023. Of these, 10 patients had GMFs and 13 SMFs. One patient in both groups had bilateral groin flaps formed. The median (range) age in the SMF and GMF groups was 67 (60–82) years and 71 (53–87) years, respectively. Demographic data are shown in Table 1. There were no statistically significant differences in patient sex, ASA, comorbidities, and indication for surgery. The mean standardised femoral artery depth was 42 mm in the SMF population and 46 mm in the GMF population. All SMFs and GMFs that were included were created in the expedited setting, as per the National Confidential Enquiry into Patient Outcome and Death (NCEPOD) criteria. The indications for intervention were pseudoaneurysm (10%) and infection (90%) in the GMF group and haematoma (8%), pseudoaneurysm (8%) and infection (84%) in the SMF group. All infections were deep surgical space infections as per the 2025 Centres for Disease Control and Prevention (CDC) Surgical Site Infection (SSI) guidelines.^21^

**Table 1:** Participant demographic characteristics.

Characteristic	Gracilis (*n* = 10)	Sartorius (*n* = 13)
Age – median	67	71
*Sex*		
Male	7	8
Female	3	5
*Ethnicity*		
Māori	4	0
Non-Māori	6	13
ASA∗	3	3
*Comorbidities*		
Type 2 diabetes mellitus	2 (20)	6 (46)
Hypertension	9 (90)	12 (92)
Ischaemic heart disease	5 (50)	11 (85)
Chronic kidney disease	1 (10)	4 (31)
Immunosuppression	0	3 (23)
*SSI class*†		
Deep	9 (90)	11 (84)
Organ space	0	0
*Indication*		
Haematoma	0	1
Pseudoaneurysm	1	1
Infection	9	11
Femoral artery depth – mm	46	42
Redo groin procedure	9 (90)	12 (92)
*In situ graft*		
PTFE	2 (20)	0
Polyester	2 (20)	5 (38)
Bovine pericardium	2 (20)	4 (31)
Native vein	3 (30)	1 (8)

Data are presented as *n* (%). PTFE = polytetrafluoroethylene.

∗ ASA – American Society of Anaesthesiologists Physical Status classification system.

† SSI class – Centres for Disease Control and Prevention (CDC) Surgical Site Infection (SSI) classification system.

### Primary outcomes

At the end of the study period, nine (69%) groins in the SMF group were completely healed, compared with nine (90%) in the SMF group (*p* = 0.34). Four patients in the SMF group compared with one in the GMF group died with an unhealed wound. The median time to complete wound healing was 55 days for SMF and 56 days for GMF. Comparison outcome data are shown in Table 2. Nine patients in the SMF group had a prosthetic graft covered by the muscle flap, of whom three (30%) did not heal their groin wound; two patients subsequently underwent a transfemoral amputation. In comparison, there were five patients in the GMF group who had a prosthetic graft left *in situ*, with all patients achieving healing without limb loss or graft occlusion.

**Table 2:** Comparison outcome data between gracilis and sartorius flaps.

Outcome	Gracilis (*n* = 10)	Sartorius (*n* = 13)	*p* value
*Death*			
30 day	2 (20)	1 (8)	1.0
12 month	1 (10)	4 (31)	0.56
*Wound healed*			0.66
30 day	3 (30)	3 (23)	1.0
12 month	9 (90)	9 (69)	0.34
*Re-intervention*			
Debridement	3 (30)	2 (15)	0.61
Flap necrosis	0	1 (8)	1.0
Graft explant	0	1 (8)	1.0
Donor site complications	2 (20)	0	0.18
Graft occlusion	0	1 (8)	1.0
Limb loss	0	2 (15)	0.48

Data are presented as *n* (%).

### Secondary outcomes

For the GMFs, three groins underwent expected re-intervention by way of further debridement. No patients developed muscle flap necrosis or needed graft explant but two patients in the GMF group needed re-intervention for haematomas at the donor site; both patients eventually healed the donor wound. By comparison, in the SMF group, two patients required expected re-intervention for ongoing infection, one patient developed muscle flap necrosis and subsequently required the underlying prosthetic graft to be explanted and ultimately underwent major limb amputation, although there were no donor site complications that required a return to the operating room. A composite outcome for flap necrosis, graft patency, graft explant, and limb loss showed no difference between the SMF (17%) and GMF (0%) groups (*p* = 0.49).

## DISCUSSION

This study presents both SMFs and GMFs from the same centre for coverage following femoral wound breakdown. In the GMF group, 90% of wounds were healed at 12 months and in the SMF group 69% were healed (*p* = 0.27). In this cohort, other than one patient who died during follow up, all patients who underwent GMFs had completely healed at the end of the twelve month period, with no associated graft or limb loss. Two patients needed a major limb amputation in the SMF group after graft loss. There were no statistically significant outcome differences between SMFs and GMFs by way of re-intervention, flap necrosis, donor site complications, and survival.

The findings in this study support the findings from a systematic review and meta-analysis of 30 studies comparing 730 SMFs, 382 RFFs, and 95 GMFs created in plastic surgery and vascular surgery patients.^22^ This review did not show any differences in complications or mortality. However, there was heterogeneity of patients in this multicentre analysis, particularly in the GMF patients, with five studies contributing 95 of the patients. Two studies previously compared SMFs and GMFs within cohorts in a single centre, a design which may account for locoregional practice and demographic homogeneity. Rajput *et al.*,^12^ compared 270 plastic surgical flaps and found SMFs and RFFs to have comparable and favourable outcomes; however, when compared with GMFs, three of the 270 flaps were GMFs. Similarly, Tanaka *et al.*^23^ compared seven SMFs with one GMF in their vascular surgery case series. These studies found 98.9% and 100% rates of healing, respectively, and infection recurrence in 39% and 38%, respectively. The present study contributed data that fit alongside these single centre cohorts; however, it had greater experience in the number of patients who underwent GMFs.

Two smaller studies have presented excellent rates of prosthetic graft salvage, limb salvage and healing in GMF coverage, which is reflected in the data.^17^^,^^24^ A separate larger cohort of 64 GMFs found complete healing in 85% patients and a limb loss rate of 19%, which was statistically significantly higher than this cohort.^16^ They found that patients with prosthetic grafts *in situ* were more prone to persistent infection and more prone to flap failure, which was not demonstrated in this cohort.

One SMF patient developed flap necrosis in this study. This patient had inflow disease with a previous prosthetic femorofemoral crossover graft. The patient subsequently developed an infected pseudoaneurysm at the femoral anastomosis and needed reconstruction with a prosthetic graft, along with an SMF for coverage. Flap failure may be attributed to poor vascular supply of the sartorius muscle by way of inflow disease, but prior studies have not adequately ascertained superficial femoral arterial supply and muscle flap success in SMF.^7^^,^^12^^,^^25^, ^26^, ^27^

A perceived benefit of the SMF is that it does not require separate incisions. In this GMF cohort, two patients developed haematomas at the donor site, which needed washout and closure in the operating room and eventually went on to heal. Both patients were current smokers and had elevated body mass indices. To reduce the risk of haematoma during GMF, the authors suggest consideration of meticulous technique with haemostasis, ensuring that all muscle feeding vessels are ligated on both sides and inserting a large bore drain (15/16 F) as routine. Harvesting the GMF from a site further away from the initial infection and scarred tissue also lends benefit, as evidenced in previous literature.^17^^,^^28^

### Limitations

One limitation of this study was the relatively small sample size, which makes interpretation of results significance difficult but reflects the nature of this very specific problem and selective use of muscle flaps. This retrospective study was conducted at a single centre, and certain variables such as body mass index data, criteria for choosing technique other than surgeon preference, and peri-operative nutritional status were not recorded, which may have introduced bias and could limit generalisability. The patients were not randomised, and the cohort was too small to carry out any meaningful propensity matching between the two muscle flap groups. Future multicentre investigation could consider these methodologies. In addition, patient reported outcomes, function, quality of life, and mobility were not captured.

### Conclusion

In instances of infected groin wound breakdown, where underlying vascular graft salvage is being undertaken, SMF and GMF are both feasible options with reasonable post-operative outcomes.

## CONFLICT OF INTEREST

There are no conflict of interests to declare.
